# VHL-dependent alterations in the secretome of renal cell carcinoma: Association with immune cell response?

**DOI:** 10.18632/oncotarget.5560

**Published:** 2015-10-12

**Authors:** Franziska Stehle, Sandra Leisz, Kristin Schulz, Nicolle Schwurack, Nico Weber, Chiara Massa, Jana Kalich, Corinna Fahldieck, Barbara Seliger

**Affiliations:** ^1^ Martin Luther University Halle-Wittenberg, Institute of Medical Immunology, 06112 Halle (Saale), Germany

**Keywords:** VHL, renal cell carcinoma, tumor microenvironment, secretome, manganese superoxide dismutase 2

## Abstract

Secreted proteins could modulate the interaction between tumor, stroma and immune cells within the tumor microenvironment thereby mounting an immunosuppressive tumor microenvironment. In order to determine the secretome-mediated, von Hippel Lindau (VHL)-regulated cross-talk between tumor cells and T lymphocytes peripheral blood mononuclear cells (PBMC) from healthy donors were either cultured in conditioned media obtained from normoxic and hypoxic human VHL-deficient renal cell carcinoma (RCC) cell line (786-0^VHL−^) and its wild type (wt) VHL-transfected counterpart (786-0^VHL+^) or directly co-cultured with both cell lines. An increased T cell proliferation was detected in the presence of 786-0^VHL+^-conditioned medium. By applying a quantitative proteomic-based approach using differential gel electrophoresis followed by mass spectrometry fourteen proteins were identified to be differentially expressed within the secretome of 786-0^VHL−^ cells when compared to that of 786-0^VHL+^ cells. All proteins identified were involved in multiple tumor-associated biological functions including immune responses. Functional studies on manganese superoxide dismutase 2 (MnSOD2) demonstrated that it was a regulator of T cell activation-induced oxidative signaling and cell death. Direct effects of soluble MnSOD2 on the growth properties and interleukin 2 (IL-2) secretion of T cells could be demonstrated underlining the critical role of extracellular MnSOD2 levels for T cell proliferation and activation.

## INTRODUCTION

Despite the recent advances in the detection as well as treatment of metastatic renal cell carcinoma (RCC) the overall 5 year's survival rate of RCC patients with metastatic disease is still low suggesting a poor prognosis and worse clinical outcome of this disease. While RCC are refractory to standard therapies and only showed an approximately 15 – 20% response rate to cytokine treatment, targeted therapies using tyrosine kinase inhibitors (TKI) have shown promising results in advanced RCC, but their clinical efficacy is limited due to the development of resistances to these drugs. Therefore, a suitable therapeutic strategy might be the combination of anti-angiogenic and immune-based treatment as first-line therapy this disease [1]. Prior to implementing such combinatorial therapeutic concepts, it is important to understand how the tumor microenvironment generates systemic immune suppression in RCC patients. Angiogenesis and immune suppression have been shown to support each other in the RCC microenvironment. The high pro-angiogenic status of RCC is associated with an accumulation of regulatory T cells (Tregs) and myeloid-derived suppressor cells (MDSC) in the local tumor microenvironment [2], in particular in the tumor invasion zone. Thus, the high frequency of Tregs and MDSC might allow RCC cells to evade immune surveillance thereby promoting disease progression [3]. The impaired anti-tumor immune response in RCC involves T cells, natural killer (NK) cells, dendritic cells (DCs), macrophages and MDSC [4, 5]. The complex interplay between various cell types of the immune system, tumor cells and the tumor microenvironment is reflected by a high dynamic range of the immune cell infiltrate as well as soluble and physical factors and therefore should be carefully accounted for the timing and selection of anti-cancer strategies, especially immunotherapy. For example, the activation of DC subpopulations at early RCC stages is counterbalanced by the co-appearance of Tregs, whereas later stages are characterized by an accumulation of neutrophils at the tumor site [6]. The presence of tumor-associated macrophages (TAMs) and high serum levels of IL-1β in RCC patients correlate with advanced disease. The presence of IL-1β in the tumor microenvironment promotes the development of aggressive RCC by inducing the expression of matrix metalloproteinase (MMP) 1, MMP3, MMP10 and MT1-MMP via activation of the transcription factor CCAAT enhancer binding protein β (CEBPβ) [7]. Macrophages that infiltrate human RCC display significantly enhanced expression levels and activity of 15-lipoxygenase-2 (15-LOX2) resulting in stimulation inflammation and immune dysfunction [8, 9].

Furthermore, the characteristics of the tissue microenvironment including metabolic changes play an integral role in supporting the proliferation of cancer cells [10, 11] suggesting that metabolic reprogramming might affect tumor growth [12, 13]. Indeed, glucose metabolism and growth control are tightly linked in proliferating cells and involve signal transduction cascades including the PI3K/Akt/mTOR pathway [14]. The “Warburg effect” describes cells exhibiting a metabolic shift toward aerobic glycolysis supporting an increased production of biomass, in particular amino acids and nucleic acids [15]. However, the metabolic signatures of cancer cells are not passive responses to damaged mitochondria, but result from oncogene-directed metabolic reprogramming required for the alterations of the composition of cellular constituents and soluble factors within the tumor microenvironment, which is accompanied by changes of the tumor growth characteristics. Recent evidence suggests that metabolites can be oncogenic by altering cell signaling and blocking cellular differentiation [13]. Moreover, the inefficient production of ATP associated with the Warburg effect results in selective advantages mediated by intracellular metabolic shifts, which have also an impact on the extracellular tumor microenvironment supporting tumor cell growth and survival [11]. This includes an increased adenosine concentration, which is involved in impaired T cell-mediated tumor rejection and support of angiogenesis [11].

Mutation, loss or methylation of both *von Hippel-Lindau* (VHL) alleles has been reported in sporadic RCC of the clear cell type (ccRCC) and in the inherited VHL syndrome. The lack of VHL protein function caused metabolic alterations, which lead to a switch from oxidative phosphorylation to aerobic glycolysis, increased glycogen synthesis along with a switch from glucose to glutamine as the major substrate for fatty acid synthesis resulting in tumor progression and therapy resistance [16]. The VHL gene product is a critical component of a multi-protein ubiquitin ligase complex targeting the regulatory hypoxia-inducible factor (HIF)-α subunits for oxygen-dependent proteolysis [17, 18]. The broad metabolic reprogramming is coordinated at the transcriptional level by HIF-1, which functions as a master regulator to balance oxygen supply and demand [16] and activates the transcription of > 100 genes involved in a variety of physiological cell processes, such as e.g. vascular endothelial growth factor (VEGF), glucose transport (glucose transporters), glycolysis (glycolytic enzymes), and cell survival (insulin-like growth factor 2) [19, 20]. Furthermore, the VHL-induced gene regulation can also occur independent of hypoxia [20, 21]: Cells defective for the VHL gene product constitutively overexpress HIF target genes irrespective of the environmental oxygen concentration [22] due to the stabilization of HIF-α subunits [23]. Using various ome-based approaches a number of studies demonstrated a differential gene and protein expression pattern in VHL^−^ and VHL^+^ RCC cells, which is partially overlapping, but also distinct from that induced by hypoxia [24–27]. However, so far it has not been determined whether and how the loss of VHL function affects the secretome of RCC cells thereby also modulating the immune cell response. In order to study the VHL-mediated changes within the tumor microenvironment a pair of RCC cell lines that are either defective or expressing the wild-type (wt) VHL gene product were used to generate conditioned media under normoxic and hypoxic conditions and in co-culture experiments with peripheral blood mononuclear cells (PBMC). The effect of the conditioned media or of the co-cultivation with VHL^−^/VHL^+^ RCC cell lines on the proliferation rate as well as the expression of distinct activation markers was determined. The results demonstrate VHL-dependent alterations of the RCC secretome, which modulate the T cell activation by negatively interfering with T cell proliferation and cytokine secretion. This could be linked to changes in the extracellular manganese superoxide dismutase (MnSOD2) concentration. Thus MnSOD2 plays an important role in the cross talk between tumor and immune cells within the tumor microenvironment of RCC.

## RESULTS

### Reduction of T cell proliferation and activation marker (CD25) expression in the presence of 786-0^VHL−^-conditioned media

**Figure 1:**
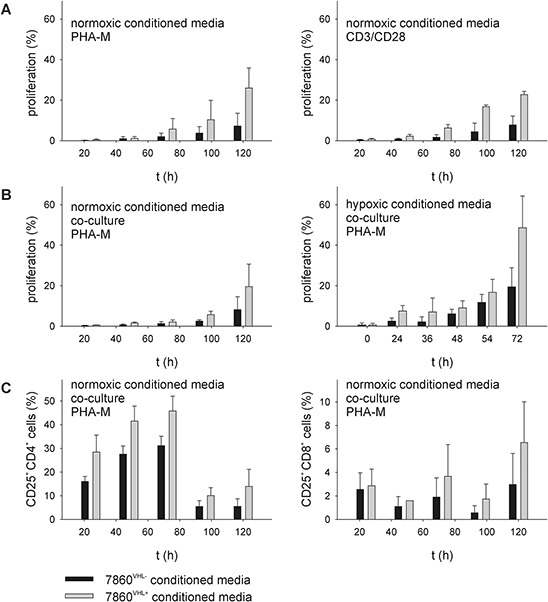
Reduction of T cell proliferation and CD25 expression within 786-0^VHL−^ conditioned media PBMC from healthy donors were prepared by Ficoll density gradient centrifugation and stimulated directly with anti-CD3 and anti-CD28 antibodies (mouse anti-human, BD, 1 μg/ml each) or PHA-M (Sigma-Aldrich, 4 μg/ml) and IL-2 (100 U/ml). “Cells were grown up to 6 days in 786- 0VHL- or 786-0VHL+ conditioned media **(A)** or in the presence of 786-0VHL-/VHL+ cells **(B)**, respectively. Cells were analyzed via flow cytometry using covalent CFSE staining (Invitrogen) according to the manufacturer's protocol.” Cells were grown for 6 days under normoxic conditions and analyzed via flow cytometry for lymphocyte markers using seven-color staining panels **(C)** Data are represented as mean percentage (%) ± SD based on five independent experiments.

To determine whether the VHL reconstitution has direct effects on CD3/CD28- or PHA-M stimulated immune effector cells, PBMC from healthy donors were cultured in media conditioned under normoxic (21% O_2_, 48 h) or hypoxic (1% O_2_, 48 h, balanced N_2_) conditions from 786-0^VHL−^ versus 786-0^VHL+^ cells and subsequently analyzed with respect to proliferation, composition and function of immune cell subpopulations. As shown in Figure 1, PBMC were analyzed by flow cytometry using covalent CFSE staining (A / B) thereby demonstrating an inhibition of cell proliferation in the presence of 786-0^VHL−^-conditioned medium. The reduced immune cell proliferation in 786-0^VHL−^ normoxic conditioned media was independent of the stimulation method used for activation (Figure 1A). The inhibition of T cell proliferation was detected in both conditioned media alone (Figure 1A) and upon co-culture with 786-0^VHL−^ cells in the pre-conditioned medium (Figure 1B). Under hypoxic conditions the effects on cell proliferation in the presence of 786-0^VHL−^ cells and 786-0^VHL−^-conditioned media were increased (Figure 1B). Furthermore, cells were grown for 72 h under normoxic conditions and analyzed by flow cytometry for the lymphocyte repertoire using seven-color staining. Flow cytometry revealed a VHL-dependent increase in the percentage of peripheral blood CD4^+^ CD25^+^ T cells within the total CD4^+^ T cell population as well as the percentage of blood CD8^+^ CD25^+^ T cells within the total CD8^+^ T cell population (Figure 1C). In contrast, other markers analyzed were not altered in dependence of VHL and hypoxia (data not shown).

### Identification of differentially secreted proteins in 786-0^VHL−^ versus 786-0^VHL+^ conditioned media

**Figure 2:**
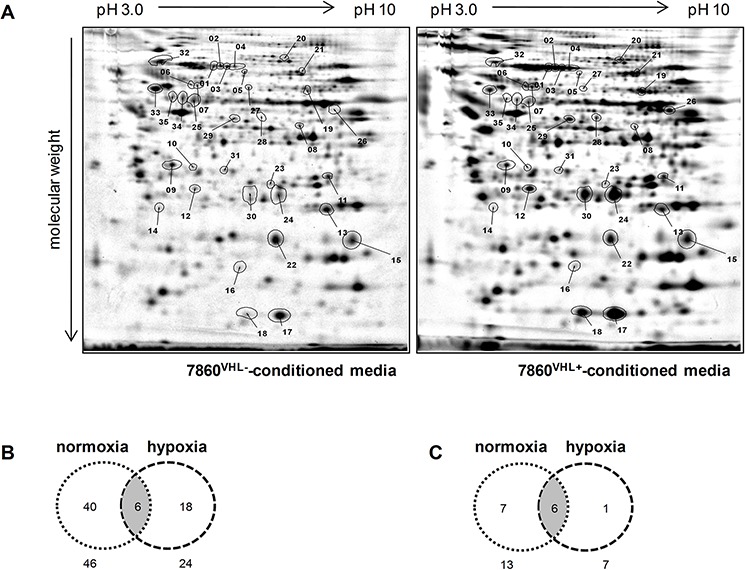
Comparative profiling of cell culture supernatants upon reconstitution of VHL expression in the 786-0 cell line **(A)** Representative protein expression profiles of media conditioned by the parental cell line 786-0^VHL−^ (left panel) and its VHL-transfected variant (786-0^VHL+^, right panel) under normoxic conditions (21% O_2_, 48 h) is shown. The respective protein pools were individually labeled with distinct fluorescence dyes (DIGE technology) and than co-separated by 2-DE as described in the Materials and methods section. Differentially expressed proteins are labeled with numbers. Figure B and C show an overview of the conditioned media profiling results. The Venn diagrams display the distribution pattern of all differentially secreted proteins under normoxic and hypoxic conditions **(B)** as well as the repeatedly detected secreted proteins **(C)**.

To determine VHL-dependent differentially secreted proteins the protein pattern of 786-0^VHL−^ and 786-0^VHL+^ conditioned media was compared by 2DE-based proteome analysis. Differentially secreted protein spots of the 786-0^VHL−^ versus 786-0^VHL+^ model system (Figure 2A) were then subjected to mass spectrometric analysis. Overall, 64 distinct proteins were found differentially secreted and identified by mass spectrometry. The vast majority of proteins (46 proteins) were differentially secreted within normoxic conditioned media of 786-0^VHL−^ versus 786-0^VHL+^ cells. 24 proteins were detected to be differentially secreted in the presence of hypoxic conditioned media of 786-0^VHL−^ versus 786-0^VHL+^ cells (Figure 2B). 6 proteins were identified upon culture in normoxic and hypoxic conditioned media, including aldose reductase (AKR1B1), cofilin-1 (CFL1), glutathione synthetase (GSS), plasminogen activator inhibitor 1 (Serpine1), superoxide dismutase (MnSOD2) and metalloproteinase inhibitor 2 (TIMP2). As listed in Table 1, 14 proteins were constantly detected as differentially secreted in 3 individual experiments (Figure 2C) upon VHL overexpression: 9 proteins were down-regulated and 5 proteins were up-regulated in conditioned media of 786-0^VHL−^ versus 786-0^VHL+^ cells.

**Table 1:** Proteins differentially secreted in media conditioned by 786-0^VHL−^- versus 786-0^VHL+^ cells

protein name	gene symbol	UniProtKB ID.	x-fold change	cellular function	cellular compartment
aldose reductase	AKR1B1	P15121	0.4 ± 0.1 ↓ **N/H**	glyceraldehyde oxidoreductase activity	cytosol, extracellular space, nucleus
retinal dehydrogenase 1	ALDH1A1	P00352	3.5 ± 1.3 ↓ **N**	aldehyde dehydrogenase (NAD) activity	cytosol
**beta- 2-microglobulin**	B2M*	P61769	2.9 ± 0.6 ↓ **N**	component of the class I major histocompatibility complex (MHC)	extracellular space, secreted
T-complex protein 1 subunit zeta	CCT6A	P40227	0.4 ± 0.1 ↓ **H**	chaperone	cytoplasm
cofilin-1	CFL1	P23528	0.4 ± 0.1 ↓ **N/H**	regulation of actin cytoskeleton dynamics	cell membrane, cytoplasm, cytoskeleton, nucleus
cathepsin D	CTSD	P07339	0.4 ± 0.1 ↓ **N**	acid protease	extracellular space, secreted, lysosome
glutathione synthetase	GSS	P48637	0.5 ± 0.1 ↓ **N/H**	glutathione biosynthesis	cytosol
matrix metalloproteinase-2 (72 kDa type IV collagenase)	MMP2	P08253	0.4 ± 0.1 ↓ **N**	metalloproteinase involved in angiogenesis, tumor invasion, inflammation	cytoplasm, extracellular matrix, membrane, mitochondrion, nucleus, secreted
**plasminogen activator inhibitor 1**	SERPINE1*	P05121	2.2 ± 0.1 ↓ **N/H**	serine protease inhibitor	extracellular space, secreted
**superoxide dismutase [Mn], mitochondrial**	MnSOD2*	P04179	0.4 ± 0.1 ↓ **N/H**	destroys superoxide anion radicals	mitochondrion
metalloproteinase inhibitor 2	TIMP2	P16035	2.7 ± 0.4 ↓ **N/H**	irreversible inactivation of metalloproteinases	extracellular space, secreted
triosephosphate isomerase	TPI1	P60174	0.4 ± 0.1 ↓ **N**	gluconeogenesis, glycolysis	cytosol
**ubiquitin-conjugating enzyme E2 N**	UBE2N*	P61088	0.5 ± 0.1 ↓ **N**	polyubiquitination, DNA repair	cytoplasm, nucleus
WD repeat-containing protein 1	WDR1	O75083	2.5 ± 0.1 ↑ **N**	induces disassembly of actin filaments in conjunction with ADF/cofilin family proteins	cytoplasm, cytoskeleton

Proteins involved in regulation of immune system processes are marked in bold.

### Functional annotation cluster analysis

**Figure 3:**
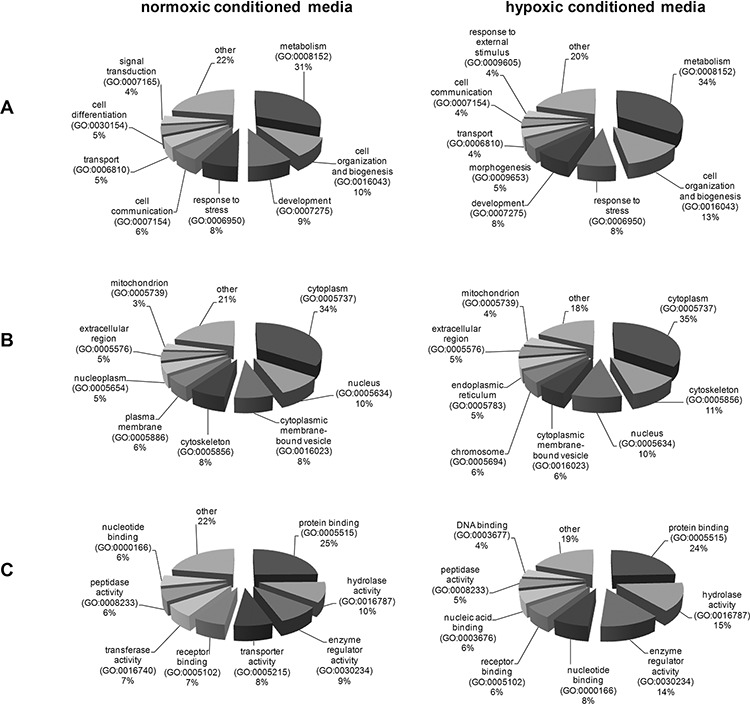
Classification of the differentially secreted proteins according to their biological processes (A), cellular components (B) and their molecular functions (C)

To categorize the biological processes that are altered in 786-0^VHL+^ conditioned media all differentially expressed proteins (fold change ≥2.0) were classified using the Functional Annotation Cluster (FAC) tool available in the Database for Annotation, Visualization and Integrated Discovery (DAVID) [http://david.abcc.ncifcrf.gov/home.jsp]. DAVID FAC analysis of 46 regulated proteins identified using normoxic conditioned media generated a total of 11 and 3 functional clusters for up-regulated and down-regulated proteins, respectively using default parameters. DAVID FAC analysis of the 24 regulated proteins identified using hypoxic conditioned media generated a total of 5 functional clusters for down-regulated proteins, respectively using default parameters. The Gene ontology (GO) terms “Biological Process”, “Cellular Component” and “Molecular Function” were used for annotations. The GO terms with highest significance and statistically significant *p*-values from the resulting functional clusters are listed in Tables 2 and 3 for hypoxic and normoxic conditioned media, respectively. Regarding there classification all identified differentially secreted proteins are involved in multiple tumor-associated biological functions and the regulation in the 786-0^VHL+^ conditioned media coincides with the reduced aggressiveness in the presence of VHL protein (Figure 3).

**Table 2:** Significantly enriched gene ontology terms detected by FAC in up-regulated and down-regulated proteins after cultivation under hypoxic conditions

	No. of AC	GO Term (fold enrichment)	No. of Proteins	*p*-value
**up-regulated**	0			
**down-regulated**	1	GO:0031988, membrane-bounded vesicle (1.98)	4	0.014
	2	GO:0031974, membrane-enclosed lumen (1.28)	6	0.021
	3	GO:0043066, anti-apoptosis (1.15)	3	0.016
	4	GO:0031012, extracellular matrix (1.14)	3	0.040
	5	GO:0006928, cell motion (0.9)	4	0.010

**Table 3:** Significantly enriched gene ontology terms detected by FAC in up-regulated and down-regulated proteins after cultivation under normoxic conditions

	No. of AC	GO Term (fold enrichment)	No. of Proteins	*p*-value
**up-regulated**	1	GO:0006986, response to unfolded protein (1.69)	4	0.00026
	2	GO:0030414, peptidase inhibitor activity (1.60)	3	0.035
	3	GO:0031988, membrane-bounded vesicle (1.44)	5	0.015
	4	GO:0031400, negative regulation of protein modification process (1.10)	3	0.019
	5	GO:0004175, endopeptidase activity (1.02)	4	0.034
	6	GO:0042981, regulation of apoptotic process (0.93)	6	0.012
	7	GO:0016192, vesicle-mediated transport (0.84)	5	0.018
	8	GO:0043161, proteasome-mediated ubiquitin-dependent protein catabolic process (0.79)	3	0.014
	9	GO:0005525, GTP binding (0.78)	3	0.016
	10	GO:0046907, intracellular transport (0.67)	5	0.027
	11	GO:0005576, extracellular region (0.65)	9	0.015
**down-regulated**	1	GO:0006732, coenzyme metabolic process (2.53)	3	0.017
	2	GO:0031988, membrane-bounded vesicle (2.48)	5	0.0064
	3	GO:0043066, anti-apoptosis (0.84)	3	0.030

### Gene ontology analysis using GOMiner

Gene ontology (GO) terms for repeatedly identified proteins were extracted, and overrepresented functional categories for differentially abundant proteins were determined by the high throughput GOMiner tool (National Cancer Institute, http://discover.nci.nih.gov/gominer/) [28]. Proteins involved in regulation of immune system processes include beta-2-microglobulin (B2M), plasminogen activator inhibitor 1 (SERPINE 1), mitochondrial superoxide dismutase (MnSOD2) and ubiquitin-conjugating enzyme E2 N (UBE2N). The proteins B2M and SERPINE 1 were up-regulated in the media conditioned by 786-0^VHL+^ cells, whereas MnSOD2 and the UBE2N were down-regulated in the respective cell culture supernatants.

### Validation of representative differentially secreted proteins in 786-0^VHL−^ versus 786-0^VHL+^ conditioned media

**Figure 4:**
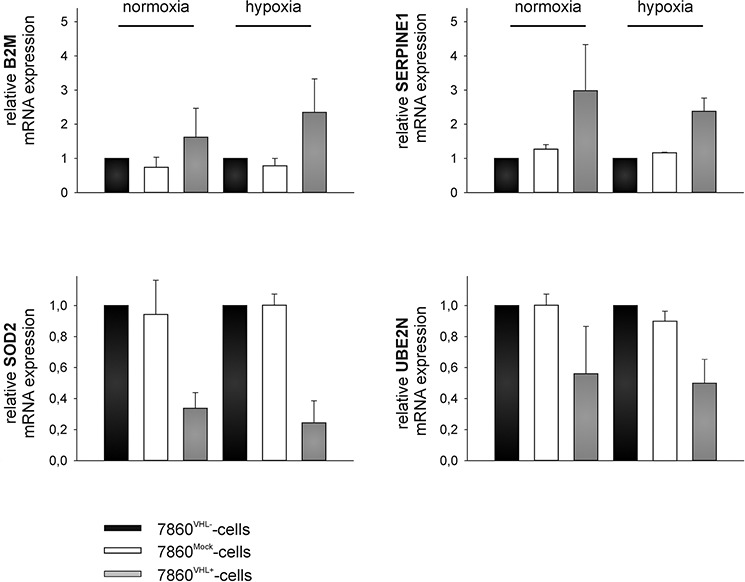
Representative qPCR-mediated target validation 786-0^VHL−^ and 786-0^VHL+^ cells were cultured under normoxic (21% O_2_, 48 h) and hypoxic (1% O_2_, 48 h) conditions and subjected to quantitative real-time PCR analysis to determine mRNA levels of B2M, MnSOD2, plasminogen activator inhibitor 1 (SERPINE1), and ubiquitin-conjugating enzyme E2 N (UBE2N) using oligo-(dT)18 primed cDNA. Mean expression of HPRT and PPIA were used for normalization. Data were represented as mean percentage (%) ± SD based on three independent experiments.

**Figure 5:**
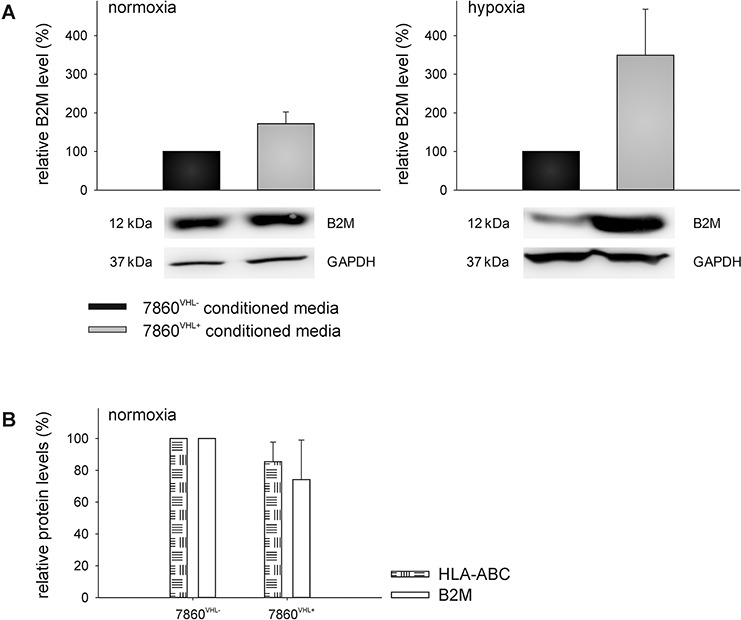
VHL-mediated up-regulation of B2M in the supernatant of 786-0VHL+ cells The up-regulation of B2M within the cell culture supernatant was verified at the protein level **(A)** as described within the Materials and Methods section. Blots were probed with an anti-B2M mAb, whereas the immunostaining with the anti-GAPDH mAb served as a loading control. In addition, the intracellular protein levels of B2M as well as HLA-ABC of 786-0^VHL−^ and 786-0^VHL+^ cells were analyzed by flow cytometry after intracellular staining using FITC-conjugated anti-B2M or FITC-conjugated anti-HLA-ABC antibodies (BD) **(B)** Data are represented as mean percentage (%) ± SD based on five independent experiments.

**Figure 6:**
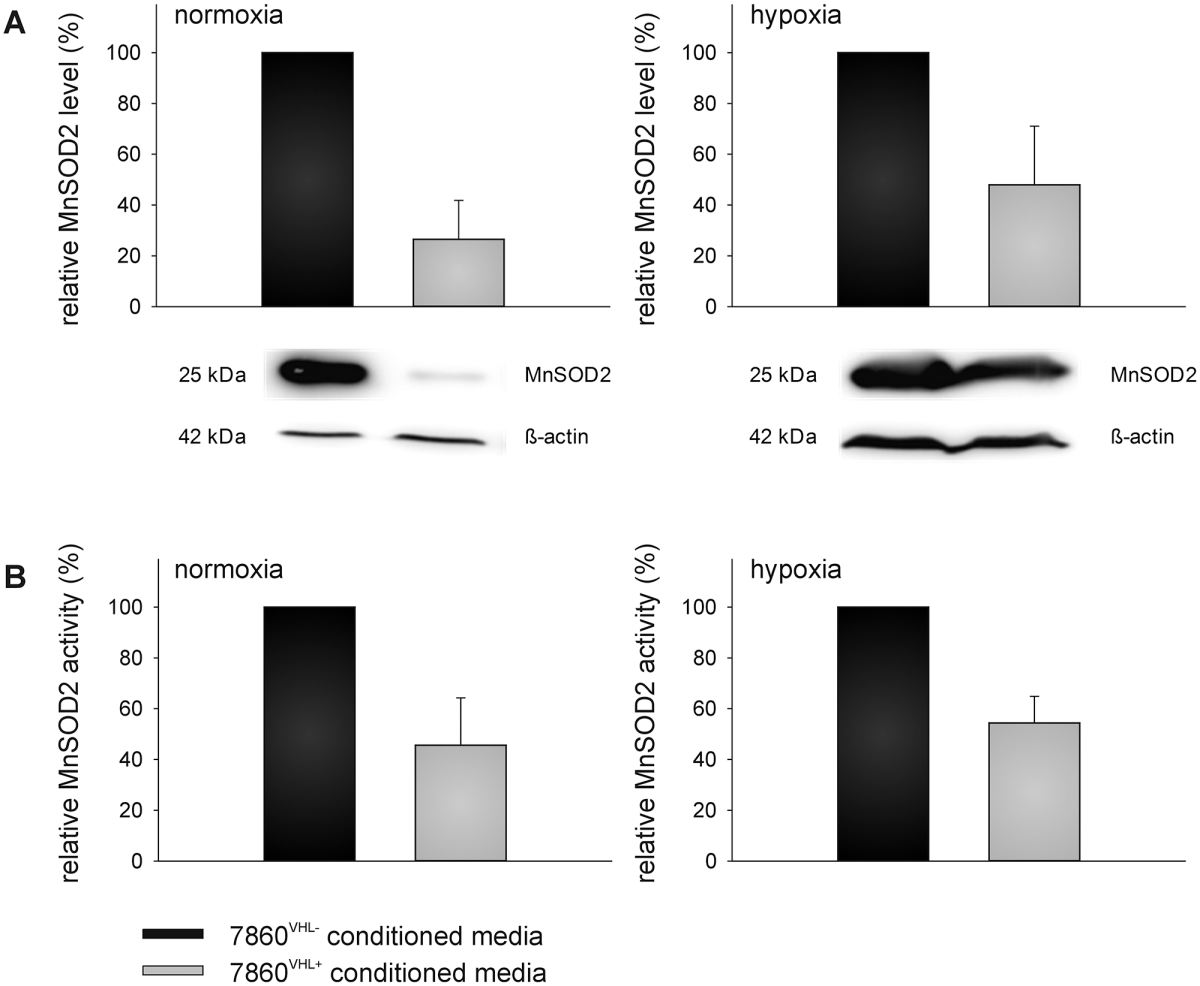
VHL-mediated down-regulation of extracellular superoxide dismutase (MnSOD2) level and activity Down-regulation of superoxide dismutase (MnSOD2) within the cell culture supernatant was also verified at the protein level **(A)** as described within the Materials and Methods section. Blots were probed with anti-MnSOD2 antibodies, whereas the immunostaining with the anti-β-actin mAb served as loading control. In addition, MnSOD2 activity was assessed using the Superoxide Dismutase Activity Colorimetric Assay Kit as described in the Materials and Methods section **(B)**.

The up-regulation of B2M and SERPINE 1 as well as the down-regulation of MnSOD2 and UBE2N in the cell culture supernatants were validated by qPCR using oligo-(dT)18 primed cDNA of 786-0^VHL−^ and 786-0^VHL+^ cells incubated for 48 h under normoxic and hypoxic conditions (Figure 4). Furthermore, the up-regulation of B2M and the down-regulation of MnSOD2 in the cell culture supernatants of 786-0^VHL+^ cells were validated by Western blot analysis using freshly prepared conditioned medium (Figure 5A and 6A). B2M is a component of the major histocompatibility complex (MHC) class I involved in the presentation of peptide antigens to the immune system. As intracellular B2M non-covalently associates with the 44 kDa heavy chain of the MHC class I antigen complex, the VHL-dependent expression of B2M and MHC class I antigens (HLA-A, B and C) was also analyzed using flow cytometry. In contrast to the VHL-dependent 1.7 - and 3.5 - fold up-regulation of B2M (*p* < 0.01) within the cell culture supernatants of 786-0^VHL+^ cells under normoxic or hypoxic conditions, respectively, no increase of intracellular B2M or MHC class I heavy chain was detectable under these conditions (Figure 5B).

With regard to MnSOD2 the supernatants of the 786-0^VHL+^ RCC cells had 3.8 − fold or 2.1 – fold lower MnSOD2 levels compared to the supernatants of the 786-0^VHL−^ cells (*p* < 0.01) under normoxic or hypoxic conditions, respectively (Figure 6A). These data were further confirmed by determination of the MnSOD2 enzyme activity demonstrating a 2.2 − fold or 1.8 – fold lower MnSOD2 activity of 786-0^VHL+^ cells when compared to the supernatants of 786-0^VHL−^ cells (*p* < 0.01) under normoxic or hypoxic conditions, respectively (Figure 6B).

### Inhibition of T cell activation-induced cell proliferation and IL-2 secretion by increased MnSOD2 levels and activity

**Figure 7:**
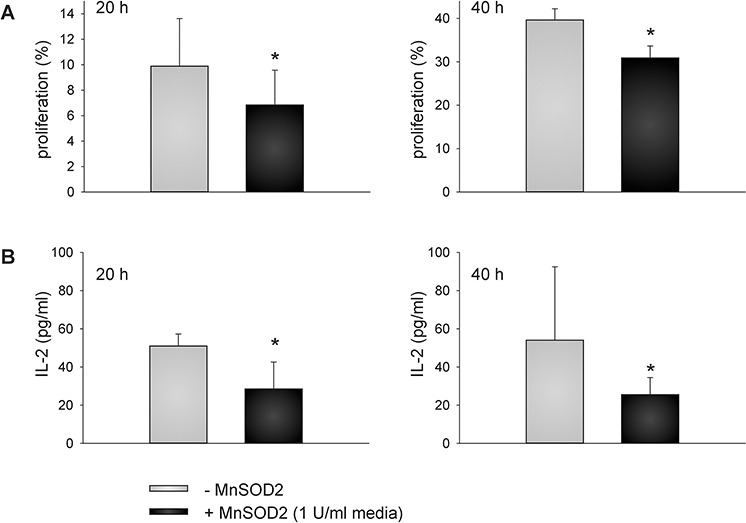
MnSOD2-induced reduction of T cell proliferation and IL-2 secretion PBMC from healthy donors were prepared by Ficoll density gradient centrifugation, labeled with CFSE according to the manufacturer 's instructions, and stimulated directly with anti-CD3 and anti-CD28 antibodies (mouse anti-human, BD, 1 μg/ml each) and IL-2 (100 U/ml). Cells were grown in the absence or presence of MnSOD2 (1U/ml) for 20 and 40 h prior to flow cytometry **(A).** IL-2 secretion was analyzed using commercial enzyme-linked immunosorbent assay (ELISA) (eBioscience, Austria) using “day 6” T cells at a density of 1 × 10^5^ cells/well onto 96-well culture plates in the absence and presence of 1 U/ml MnSOD2 as described in Materials and Methods section **(B).** Data are represented as mean percentage (%) ± SD based on five independent experiments. *p-value < 0.05

To determine whether the reduced MnSOD2 secretion as a consequence of the VHL reconstitution caused an increased proliferation of stimulated immune effector cells within the supernatants of 786-0^VHL+^ cells, PBMC from healthy donors were stimulated with anti-CD3 and anti-CD28 antibodies in the absence and presence of recombinant MnSOD2 protein followed by the analysis of their proliferation capacity using CFSE staining. As shown in Figure 7A, an inhibition of cell proliferation in the presence of MnSOD2 protein was detected, which was comparable to the diminished immune cell proliferation in the presence of MnSOD2 within the supernatants of 786-0^VHL−^ cells.

To prove whether the reduced cell proliferation rate could be linked to MnSOD2-mediated alteration of cytokine secretion, the IL-2 concentration was determined using a commercial ELISA assay. Therefore, “day 6” T cells were re-stimulated with anti-CD3 and anti-CD28 antibodies in the absence and presence of recombinant MnSOD2 protein for 20 and 40 h and their IL-2 secretion was subsequently analyzed (Figure 7B). The supernatants of MnSOD2-treated re-stimulated “day 6” T cells showed 1.8 and 2.1 – fold lower IL-2 levels after 20 and 40 h of re-stimulation, respectively when compared to the supernatants lacking recombinant MnSOD2 (*p* < 0.01).

## DISCUSSION

To improve treatment of RCC using combinations of anti-angiogenic and immune-based therapy an increased knowledge of the interactions between tumor, stroma and distinct immune cell types are necessary. As secreted proteins are supposed to be involved in the interaction amongst these cells within the tumor microenvironment the VHL-regulated cross talk between tumor cells and T lymphocytes and the involvement of the secretome in this process was analyzed in the present study.

To identify differentially secreted proteins in 786-0^VHL−^ versus 786-0^VHL+^ conditioned media responsible for reduction of T cell proliferation and activation marker (CD25) expression within 786-0^VHL−^ conditioned media (Figure 1), the protein pattern of 786-0^VHL−^ and 786-0^VHL+^ conditioned media was compared using 2DE-based proteome analysis. The secretome study was performed *in vitro* by first culturing 786-0^VHL−^ and 786-0^VHL+^ cells in serum-supplemented medium to obtain a sufficient number of cells. Subsequently cells are washed carefully with sterile phosphate-buffered saline to remove bovine serum proteins followed by incubation in serum-free medium up to 72 hours. Conditioned medium containing cell-secreted proteins was collected and analyzed by 2D gel electrophoresis prior to protein identification by mass spectrometry.

Altogether, 64 proteins were found differentially secreted in dependence of the VHL status from which 14 proteins were constantly found in 3 individual experiments (Figure 2; Table 1).

The relative low number of recurring proteins might be explained by false-positive discoveries due to serum proteins or proteins released by dying cells or as the result of mechanical injury [29]. The incubation of mammalian cells in serum-free medium to avoid interference from serum contaminants might affect the cell secretome profile. In addition, the length of incubation in serum-free medium could also dramatically influence the secretome profiles, as increased cell death correlates with an increased release of intracellular proteins [29].

The functional annotation cluster analysis of all identified proteins clearly showed an enrichment of proteins localized within membrane-bounded vesicles or in the extracellular region (Table 2 and 3; Figure 3). Furthermore, intracellular proteins like anti-apoptotic proteins or proteins of the unfolded protein response were additionally identified (Table 2 and 3).

Several intracellular proteins are consistently found to be released by cultured mammalian cells into the conditioned medium. Although these intracellular proteins can be released due to cell death or leakage, there is evidence that some of these proteins are secreted via non-classical pathways like vesicles and exosomes [30]. Often these proteins possess additional extracellular functions that are different from their intracellular role. Exosomes are small vesicles with an average size ranging from 30 to 150 nm. They are believed to originate from endosomes and contain a variety of components including mRNA, miRNA and proteins such as carbohydrate and lipid metabolism enzymes, cytoskeletal components and chaperone proteins [31, 32]. Exosomes might play a role in protein turnover, serve as carriers for RNA and miRNA as well as modulate the immune response [33]. The enrichment of proteins that are localized within membrane-bounded vesicles clearly showed the involvement of exosome release for modification of the cell culture supernatants of 786-0^VHL−^ and 786-0^VHL+^ cells. For example, α-enolase (ENO1), a glycolytic enzyme, involved in the synthesis of pyruvate, was detected in significant amounts in the secretome of almost every type of cultured mammalian cell. Additionally, it was down-regulated within the supernatant of 786-0^VHL+^ cells (regulation factor 0.4). Extracellular or cell surface ENO1 was suggested to act as a plasminogen receptor mediating extracellular matrix degradation and cell migration in cancer and to have diagnostic and prognostic values [34]. Down-regulation of ENO1 within the supernatant of 786-0^VHL+^ cells corresponds with the reduced aggressiveness of the 786-0^VHL+^ cells.

The 14 proteins, which were constantly found regulated in dependence of VHL in 3 individual experiments, were analyzed with respect to their GO terms [28]. The proteins B2M and SERPINE 1 were up-regulated in the media conditioned by 786-0^VHL+^ cells, whereas MnSOD2 and the UBE2N were down-regulated in this cell culture supernatant. The VHL-dependent down-regulation of MnSOD2 as well as the upregulation of B2M was further validated by qPCR, Western Blot analysis as well as MnSOD2 activity assays (Figure 4 and 6). In contrast to MnSOD2, B2M protein was just found up-regulated within the cell culture supernatants of 786-0^VHL+^ cells and not intracellular (Figure 5).

RCC comprises a heterogeneous group of tumors with various molecular and cytogenetic abnormalities and different histological features as cell types and tumor architecture [35]. Untargeted metabolomic analyses of murine ccRCC model demonstrated that HIF1α activates the transcription of genes that cause increased glucose uptake, glycolysis, and lactate production. In addition, it diminishes the flux of pyruvate entering the tricarboxylic acid cycle accompanied with a decreased oxidative phosphorylation identical to those observed in human ccRCC samples [36]. Furthermore, molecular genetic and proteomic tools led to the discovery of potential diagnostic, prognostic and therapeutic biomarkers of this disease. In early studies, the 2-DE expression patterns from whole renal and RCC tissues resulted in a number of differentially expressed proteins associated with RCC. This includes MnSOD2, a mitochondrial enzyme related to the redox cycle, involved in various regulatory functions of cells, which was not present in normal kidney tissue [35, 37, 38]. It is noteworthy that there exists a large diversity of MnSOD2 levels in various human cancers, which is most likely due to the combination of heterogeneity of genomic events and environmental changes [35].

MnSOD2 is an anti-oxidative enzyme responsible for the defense against O_2_^−^ radicals released by the electron chain as a byproduct of respiration. Moreover, MnSOD2 plays a key role in the regulation of human T cell activation since it mediates the first step in the mitochondrial oxidative signal generation. Thus, MnSOD2 represents an important control switch in the process of activation-induced oxidative signal generation in T cells.

The TCR-mediated gene expression strictly depends on the simultaneous activation of oxidation-dependent and calcium-dependent transcription factors. The activation-induced oxidative signal is generated by different enzymes including the NADPH oxidases NOX2 and DUOX1 [39, 40]. Since the mitochondrial respiratory complex I was identified as an essential source of reactive oxygen species (ROS) generation upon TCR stimulation mitochondria could function as oxidative signaling organelles during T cell activation [41, 42]. After the complex I-mediated formation of O_2_^−^ radicals, the transformation into the oxidative signaling molecule H_2_O_2_ occurs within the mitochondrial matrix [43]. H_2_O_2_ diffuses into the cytosol and potentiates TCR signaling via reversible inactivation of negative regulatory phosphatases or direct influence on NF-κB/AP-1 activity or DNA binding occurs [44–46]. In order to avoid harmful ROS effects as well as to allow signaling integration, the TCR-induced generation of mitochondrial oxidative signals has to be tightly regulated. Following T cell activation the mitochondrial complex I-mediated release of O_2_^−^ radicals is up-regulated, while MnSOD2 participates in generation of mitochondrial H_2_O_2_ during the signaling phase. In the presence of low MnSOD2 levels, H_2_O_2_ could also be produced by reactions other than O_2_^−^ radical dismutations, which have substrate/product ratios higher than MnSOD2. The resulting increased cytoplasmic H_2_O_2_ levels activate the redox-dependent transcription factors NF-κB- and AP-1. As a consequence the expression of CD95L, IL-2 and MnSOD2 is initiated. The transcriptional upregulation of MnSOD2 results in higher mitochondrial concentrations and activity leading to the inhibition of H_2_O_2_ release. The reduced probability of O_2_^−^ radicals to participate in other H_2_O_2_-generating reactions yielding more H_2_O_2_/single O_2_^−^ radical than the dismutase reaction itself lowers cytoplasmic H_2_O_2_ concentrations resulting in a decreased NF-κB/AP1 activation [47–49].

In this context it was shown that the TCR-induced oxidative signal generation as well as the NF- κB/AP1-dependent IL-2 gene expression inversely correlates with the protein content of MnSOD2 [48]. Consequently, MnSOD2 over-expression abrogated T cell activation-triggered mitochondrial ROS production as well as NF-κB/AP-1-mediated transcription [48]. In line with this report the proliferation of TCR-stimulated immune effector cells was significantly inhibited in the presence of recombinant MnSOD2 protein (*p* < 0.01) (Figure 7A). Therefore, the VHL-mediated increase of cell proliferation of stimulated immune effector cells within the supernatants of 786-0^VHL+^ cells could at least partly be attributed to decreased extracellular MnSOD2 levels and activity. IL-2 secretion of TCR-stimulated T cells was also slightly, but significantly diminished in presence of recombinant MnSOD2 protein (Figure 7B). Upon the internalization into the mitochondria the increased amount of extracellular MnSOD2 remove O_2_^−^ radicals more efficiently thus mimicking the inhibition of the signal transduction. Consequently, lowered expression of IL-2 resulted in decreased IL-2 secretion in the presence of recombinant MnSOD2 (Figure 7B). Our data underline the critical role of MnSOD2 in T cell response and suggest that MnSOD2 could represent a novel target for (immuno-) therapies.

## MATERIALS AND METHODS

### Chemicals

Recombinant MnSOD2 protein was purchased from Abnova (Pforzheim, Germany). Endotoxins were removed using the Detoxi-Gel™ Endotoxin Removing Gel (Thermo Fisher Scientific, Rockford, IL, USA) according to the manufacturer's instructions prior to sterile filtration using a 0.22 mm syringe filter. MnSOD2 activity was analyzed using the Superoxide Dismutase Activity Colorimetric Assay Kit (Abcam, Cambridge, UK) according to the manufacturer's protocol.

### Cell culture and preparation of conditioned media

The parental ccRCC cell line 786-0 harboring a mutated *VHL* gene (786-0^VHL−^) and its counterpart stably transfected with a HA-tagged wt VHL gene construct (786-0^VHL+^) have been recently described [26]. Both cell lines were grown in Dulbecco's modified Eagle medium (DMEM; Sigma-Aldrich, Munich, Germany) supplemented with 10% fetal calf serum (FCS, PAA Laboratories GmbH, Coelbe, Germany), 2 mM L-glutamine (Lonza, Cologne, Germany), 1% sodium pyruvate (GIBCO, Life technologies, Carlsbad, USA), 1% non-essential amino-acid mixture (GIBCO), and 1% penicillin/streptomycin (PAA).

To prepare conditioned media 1 × 10^6^ parental 786-0^VHL−^ or 786-0^VHL+^ RCC cells, respectively, were seeded in complete medium under conventional conditions (37°C, humidified atmosphere, containing 5% CO_2_) for 24 hours. Cells were then washed 3 times with phosphate buffered saline (PBS) before the medium was changed into an FCS-free Roswell Park Memorial Institute Medium (RPMI-1640; Sigma-Aldrich) supplemented with 2 mM L-glutamine (Lonza), 1% sodium pyruvate (GIBCO), 1% non-essential amino-acid mixture (GIBCO), and 1% penicillin/streptomycin (PAA) and exposed to normoxia (21% O_2_, 5% CO_2_) or hypoxia (1% O_2_, 5% CO_2_, and balanced N_2_) for 48 h. In addition, the apoptosis rate of the cells grown under normoxia and hypoxia was determined. Conditioned medium of non-apoptotic cells (apoptosis <5%) was collected, centrifuged at 300 × *g* for 5 min and at 5000 × *g* for 10 min, and finally filtered using a 0.22 mm syringe filter.

PBMC of healthy donors obtained from the local Department of Transfusion Medicine were isolated from heparinized venous blood samples by density gradient centrifugation using Leukosep^®^ (Greiner Bio-One, Germany) and Biocoll Separation Solution (Biochrome, Berlin, Germany). PBMC were either cultured under conventional conditions (37°C, humidified atmosphere, containing 5% CO_2_, 72 h) in conditioned media supplemented with 10% FCS (PAA) in the presence of 100 U/ml IL-2 (PROLEUKIN, Chiron, Ratingen, Germany) or co-cultured with 786-0^VHL−^ or 786-0^VHL+^ cells in the presence of conditioned media and directly stimulated with plate-bound agonistic anti-CD3 and anti-CD28 antibodies (mouse anti-human, 1 μg/ml each, BD Biosciences, Heidelberg, Germany) or PHA-M (Sigma-Aldrich). For co-culture experiments 2 × 10^6^ PBMC/well were seeded onto six-well culture plates and incubated with conditioned media for 120 or 72 hours under normoxic or hypoxic conditions, respectively. 786-0^VHL−^ or 768–0^VHL+^ cells were added at a density of 3 × 10^5^ on 25-mm Anopore tissue culture plate inserts (0.02 μm pore size; Nalge Nunc International, Thermo Fisher Scientific).

T cells were isolated from PBMC after labeling with allophycocyanine (APC)-conjugated anti-CD3 antibodies using the EasySep™ Human APC Positive Selection Kit according to the manufacturer's instructions (Stemcell Technologies, Grenoble, France). T cells were expanded by stimulation with plate-bound anti-CD3 and anti-CD28 antibodies (mouse anti-human, 1 μg/ml each) and subsequent culture in IL-2-containing (25 U/ml, PROLEUKIN) RPMI-1640 (Sigma-Aldrich) supplemented with 10% FCS (PAA) and 2 mM L-glutamine (Lonza) for 5–6 days (“day 6” T cells).

### Proliferation assay

To evaluate cell proliferation, 1 × 10^7^ PBMC in 10 ml of PBS were labeled with 5,6-carboxyfluorescein succinimidyl ester (CFSE, 0.5 μM, Invitrogen, Eugene, USA) according to the manufacturer's instructions. 2 × 10^6^ cells/well were seeded in 6 well microtiter plates (TPP, Trasadingen, Switzerland) in 5 ml of 786-0^VHL−^ or 786-0^VHL+^ conditioned media or in co-culture with 3 × 10^5^ 786-0^VHL−^ / 786-0^VHL+^ cells/well in the presence of conditioned media followed by direct stimulation with plate-bound anti-CD3 and anti-CD28 antibodies (mouse anti-human, 1 μg/ml each, BD Biosciences) and maintained for 5 days in culture. 10,000 events were analyzed on a BD FACSCanto II flow cytometer in combination with the FACSDiva software package (BD Biosciences) in a time kinetic. The results were expressed as percentage of proliferating cells.

Proliferation assays in the presence of MnSOD2 were analyzed as described above in the absence and presence of 1U/ml recombinant MnSOD2 (Abnova; Pforzheim, Germany) within the culture medium for up to 40 hours.

### Monitoring VHL-dependent alterations in the composition and function of T cell subpopulations

Seven-color flow cytometric staining was used for analyzing VHL-dependent effects on CD3/CD28-stimulated PBMC. Briefly, 2 × 10^6^ cells/well were seeded in 6 well microtiter plates (TPP) in 5 ml of 786-0^VHL−^or 786-0^VHL+^ conditioned media or in co-culture with 3 × 10^5^ 786-0^VHL−^ / 786-0^VHL+^ cells/well in the presence of conditioned media followed by direct stimulation with plate-bound anti-CD3 and anti-CD28 antibodies (mouse anti-human, 1 μg/ml each, BD Biosciences). After 68 h, BD GolgiPlug™ (5 μl) and IL-2 (100 U/ml) were added to each cell suspension and further incubated for 4 h. The cells were harvested and washed twice with PBS. Surface molecules were directly stained with the fluorescence-conjugated antibodies against CD3, CD4, CD8, CD25 and CD45 as well as the respective fluorescence-labelled lgG controls (all purchased from BD Biosciences) for 15 min at room temperature. Cells were washed with PBS and fixed in PBS containing 1% paraformaldehyde. Three independent experiments were performed.

### Apoptosis assay

To evaluate the VHL-mediated apoptosis induction, 2 × 10^6^ PBMC were cultured in 5 ml of 786-0^VHL−^or 786-0^VHL+^ conditioned media or in co-culture with 3 × 10^5^ 786-0^VHL−^ / 786-0^VHL+^ cells/well in the presence of conditioned media followed by direct stimulation with plate-bound anti CD3-and anti-CD28 antibodies (mouse anti-human, 1 μg/ml each, BD Biosciences) for 72 h. Apoptosis was determined by flow cytometry after staining of cells with APC-annexin V (BD Phamingen™) and propidium iodide (2 mg/ml, Sigma-Aldrich) according to the manufacturer 's instructions (BD Phamingen™). The stained cells were analyzed using the BD FACSCanto II flow cytometer and the FACSDiva software package (BD).

### cDNA synthesis and qPCR

Total RNA was extracted from the samples using the Nucleospin Extract II kit (Macherey-Nagel, Düren, Germany) according to the manufacturer's instructions. cDNA was synthesized from 3 μg RNA treated with DNase I (Invitrogen GmbH, Karlsruhe, Germany) using oligo dT primers (Fermentas, Mannheim, Germany) and the RevertAid™ H Minus First Strand cDNA synthesis kit (Fermentas, St. Ingbert, Germany) before qPCR was performed with target-specific primers (Table S1) using Platinum^®^ SYBR^®^ Green qPCR SuperMix-UDG (Invitrogen) and applying the following parameters for 40 cycles; denaturation 95°C, 15 s; annealing/elongation 60°C, 30 s. Relative mRNA expression levels for specific genes were normalized to peptidylprolylisomerase A (PPIA) and hypoxanthine-guanine phosphoribosyl-transferase (HPRT). The transcription levels of 786-0^VHL−^ cells were set to one and the relative expression ratios in 786-0^VHL+^ cells were calculated, respectively.

### NaDOC/TCA precipitation

Proteins of the cell culture supernatants were precipitated using the sodium deoxycholate (NaDOC)/TCA precipitation procedure described by Bensadoun and Weinstein [50], which allows a quantitative recovery of protein samples [51].

The samples were mixed with 10% aqueous NaDOC (1:100, by volume). Then 50% aqueous TCA (1:20, by volume) was added. After incubation at room temperature for 15 min the samples were centrifuged in 50 ml centrifuge tubes (Greiner) at 4,500 *g* and 4°C for 45 min. The supernatant was decanted and the pellet was washed with 20 ml aqueous ethanol (70%). After incubation at −20°C for 15 min, the samples were centrifuged at 4,500 *g* and 4°C for 45 min. The supernatant was decanted and 1 ml aqueous ethanol (70%) was added to the pellet followed by thorough mixing. Then the pellet was transferred into a 1.5 ml cup (Eppendorf). After incubation at −20°C for 15 min, the samples were centrifuged at 21,000 *g* and 4°C for 15 min. The supernatant was decanted and 1 ml aqueous acetone (80%) was added to the pellet followed by thorough vortexing. After incubation at −20°C for 15 min, the samples were centrifuged at 21,000 *g* and 4°C for 15 min. The supernatant was decanted and the pellet was dried under nitrogen.

### Resolubilization of protein pellets

The precipitates of 1 I cell culture supernatant/conditioned media were incubated in 200 μl DIGE lysis buffer (30 mM Tris–HCl buffer, pH 8.5, containing 7 M urea (Applichem, Darmstadt, Germany), 2 M thiourea (Sigma-Aldrich) and 4% (w/v) 3 [(3 cholamidopropyl)-dimethylamino] 1 propane sulfonate (CHAPS; Applichem)) at 25°C for 30 min thereby vortexing thoroughly. Solubilized proteins were then sonicated using two cycles of five impulses (0.5 s/impulse) at 100% power (Bandelin UW 2070 sonicator, MS 73 needle; Bandelin, Berlin, Germany) and total protein concentration of samples was determined as previously described [52].

### Fluorescence labeling of protein pellets

All fluorescence labelings were performed in DIGE lysis buffer using the *Refraction-2D Labeling Kit* according to the manufacturer's instructions (NHDyeAGNOSTICS, Halle (Saale), Germany). Briefly, 25 μg of a mixture composed of equal amounts of proteins precipitated from 786-0^VHL−^- and 786-0^VHL+^-conditioned media were labeled for 30 min on ice with 100 pmol of G-Dye 100 as internal protein standard, whereas 25 μg of individual protein representing either proteins of 786-0^VHL−^- or 786-0^VHL+^-conditioned media were independently labeled with 100 pmol of G-Dye 200 and −300, respectively. The individual labelings were performed in a total volume of 21 μl DIGE lysis buffer, respectively for 30 min on ice. The reaction was stopped by adding 1 μL 10 mM lysine. After an incubation on ice for 10 min, an equal amount of 2 × sample buffer (7 M urea, 2 M thiourea, 0.4M NDSB-256 (Merck Biosciences, Darmstadt, Germany), 4% CHAPS, 2% dithiothreitol (DTT; Applichem), 1% pharmalytes pH 3–10 (Amersham Biosciences, Freiburg, Germany) was added to the mixture before the samples were pooled and subjected to 2-DE separation.

### Two-dimensional gel electrophoresis (2-DE), protein visualization and image analysis

For analytical gels the volume of the labeled protein mix was adjusted with Lysis-buffer (7 M urea, 2 M thiourea, 0.2 M dimethylbenzylammonium propane sulfonate (NDSB 201, Merck, Darmstadt, Germany), 1% dithiothreitol (DTT; Applichem), 4% 3 [(3 cholamidopropyl)-dimethylamino] 1 propane sulfonate (CHAPS; Applichem), 0.5% pharmalytes (Amersham Biosciences, Freiburg, Germany) and a trace of the dye bromophenol blue (Serva Electrophoresis, Heidelberg, Germany) to a final volume of 350 μl, whereas for preparative gels 500 μg protein in a total volume of 350 μl lysis buffer were loaded. Samples were applied to IPG strips (pH 3–10 NL, 18 cm, GE Healthcare, Munich, Germany) by in-gel rehydration and covered with 450 μl Immobiline DryStrip Cover Fluid (GE Healthcare). After 2 h of rehydration, isoelectric focusing (IEF) was carried out at 20°C on an Ettan IPGphor 2 unit (GE Healthcare) at the following settings: 30 V 10 hrs, 500 V 1 h, 1000 V 1 h, 5000 V 1 h, and 8000 V up to a total of 45,000 Vhrs. The IPG strips were subjected to a 2 step strip equilibration procedure, which was performed by incubation the strips first for 15 min in 12 ml equilibration buffer I (6 M urea, 2% SDS, 50 mM Tris HCl (pH 8.8), 30% glycerol, supplemented with 1.5% DTT) followed by 15 min in 12 ml equilibration buffer II (6 M urea, 2% SDS, 50 mM Tris HCl (pH 8.8), 30% glycerol, supplemented with 4.8% iodoacetamide, all chemicals purchased from Applichem). SDS-PAGE separation was performed using a PROTEAN plus Dodeca Cell (Bio-Rad, Munich, Germany) with gels of 1.5 mm thickness and an acrylamide/bisacrylamide matrix of 2.5% C and 13% T (Serva, Heidelberg, Germany). Strips were fixed on vertical SDS-PAGE gels with 1.5% soft melting agarose (BioLine GmbH, Luckenwalde, Germany) and traces of bromophenol blue. Electrophoresis was performed with constant voltage (20 V, 1 h; 120 V, 15 h) at 10°C.

Gel documentation was accomplished by using a Fuji FLA 5100 fluorescence-scanning device (Fuji Photo Film, Duesseldorf, Germany). The PMT for all scans was set to 50 V below the saturation point of the brightest spot and all gel images were acquired at a resolution of 100 μm.

After 2D separation the preparative gels were stained with colloidal Coomassie Blue staining solution (10% ammonium sulfate (Applichem), 10% phosphoric acid (Merck KGaA), 0.12% Coomassie Brilliant Blue G250 (Applichem), 20% methanol (Merck KGaA); [53], and thereafter destained by extensive washing in ddH_2_O. Preparative gels were scanned on a conventional scanner (UMAX Image Scanner, GE Healthcare) at a resolution of 600 dpi and stored as TIFF-images.

2D gel image analysis was performed using Delta2D Software version 4.0.8 (DECODON GmbH, Greifswald, Germany). All gel images were matched with the Delta2D software and a synthetic fusion gel was prepared. Final spot detection was performed on the fused gel. The resulting spot pattern was assigned to each of the gels in the experiment. The normalization procedure of the Delta2D software for DIGE experiments is based on the internal protein standard (IPS). The absolute spot volume is divided by the accumulated absolute volume of all spots and subsequently normalized to the corresponding absolute spot volume of the IPS. Furthermore, Student's *t*-test was performed to assess the statistical significance of differentially expressed proteins. Spots whose relative expression is changed at least 2-fold (increase or decrease) at 95% confidence level (*t*-test; *p* < 0.05) were considered to be significant and subsequently subjected to further analysis.

### In-gel digestion and MS

Digestion with trypsin and subsequent spotting of peptide solutions onto the MALDI-targets were performed as previously described [52] with slight modifications. For protein identification the proteins were excised from Colloidal Coomassie Brilliant Blue stained 2D gels using the Herolab spot hunter (Herolab GmbH, Wiesloch, Germany) with a picker head of 1.5 mm diameter and destained by addition of 50% (v/v) ACN. Gel pieces were washed twice with 100 μl 50% (v/v) ACN and once with 100 μl 100% ACN. After drying 5 μl trypsin solution containing 17 ng/μl trypsin (Promega, Madison, WI, USA) in 25 mM NH_4_CO_3_ supplemented with 0.4 mM CaCl_2_ was added and incubated on ice for 2 h followed by an incubation over night at 37°C. If necessary 5 μl of 25 mM NH_4_CO_3_ supplemented with 0.4 mM CaCl_2_ were added to keep gel pieces hydrated throughout the digest. Gel pieces were agitated by sonicating in a water bath for 10 min before 1 μl of the supernatant (containing tryptic peptides) were mixed with 1 μl of alpha-cyano-4-hydroxycinnamic acid (HCCA) matrix (saturated at room temperature in 50% ACN, 0.1% TFA) and 1 μl of this solution were directly spotted on the MALDI-target. Prior to the measurement the samples were allowed to dry on the target.

Spectra were calibrated externally using Peptide calibration standard II (Bruker Daltonics Inc, Bremen, Germany). MALDI-TOF-MS was performed on an *ultrafleXtreme*™ mass spectrometer (Bruker Daltonics Inc) in positive reflector mode using an accelerating voltage of 25 kV. Spectra processing was performed with flexAnalysis (3.3.80.0) software for resolution-based peak detection using default settings.

The PMF dataset were analyzed using the MASCOT search engine (http://www.matrixscience.com) with the following parameters: (i) *homo* sapiens sequences; (ii) fixed modification, carbamidomethylation of cysteins; (iii) cleavage enzyme, trypsin; (vi) a maximum of one missed cleavage was allowed; and mass tolerance (monoisotopic), ± 50.0 ppm. Target identification was based on the overall sequence coverage of matching peptide fragments. Proteins were assigned when the MASCOT score exceeded 57 according to the MASCOT-defined significance threshold for false-positive events at *p* < 0.05.

The heterogeneous set of the identified significant differentially expressed proteins was analyzed using gene ontology (GOminer) software [28], which provides information about gene function and cellular localization.

### Functional annotation cluster and pathway analysis DAVID

Functional annotation cluster analysis was performed on the list of up-regulated and down-regulated proteins with a fold change of ≥2.0 [http://david.abcc.ncifcrf.gov/home.jsp]. Only those terms that reported a *p*-value of ≤ 0.05 were selected for analysis. The Gene Ontology (GO) terms of cellular components, molecular function and biological processes in DAVID were employed to categorize enriched biological themes in up- and down-regulated protein lists.

### Western blot analysis

Aliquots of 50 μg of solubilized protein/lane were separated on 12% SDS-PAGE gels and subsequently transferred onto nitrocellulose membranes (Schleicher & Schuell, Dassel, Germany). Membranes were processed as previously described [54] using target protein-specific primary antibodies directed against beta-2-microglobulin (B2M) (kindly provided by Dr. Soldano Ferrone; [55]), MnSOD2 (Abfrontier, Aachen, Germany), GAPDH (Cell signaling) or β-actin (Sigma-Aldrich) in combination with horse-radish peroxidase (HRP)-conjugated secondary antibodies (Cell Signaling, Frankfurt, Germany). Protein bands were visualized with LumiLight Western Blotting substrate (Roche, Mannheim, Germany) and recorded with a LAS 3000 CCD camera system (FUJIFILM, Duesseldorf, Germany). The co-detection of the GAPDH or β-actin signal, respectively, for each lane on the given blot served as loading control and the relative protein expression level for each target were defined using AIDA software (Raytest, Sprockhoevel, Germany).

### MnSOD2 activity assay

MnSOD2 activity of conditioned media was analyzed using the Superoxide Dismutase Activity Colorimetric Assay Kit (Abcam, Cambridge, UK) according to the manufacturer's protocol using freshly prepared and undiluted 786-0^VHL−^- and 786-0^VHL+^-conditioned media. MnSOD2 activity was analyzed after incubation up to 60 min using a microplate reader (MRX-TC, DYNEX Technologies, Denkendorf, Germany). The absorbance values were expressed as percentage of the supernatant of 786-0^VHL−^ cells.

### Determination cell proliferation and IL-2 secretion in the absence and presence of MnSOD2

IL-2 secretion was analyzed using commercial enzyme-linked immunosorbent (ELISA) assay (eBioscience, Austria) based on the supplier's protocol. Therefore, “day 6” T cells were plated at a density of 1 × 10^5^ cells/well onto 96-well culture plates in 200μl of RPMI-1640 (Sigma-Aldrich) supplemented with 10% FCS (PAA) and 2 mM L-glutamine (Lonza) in the absence and presence of MnSOD2 (1U/ml, Abnova via Biozol, Eiching, Germany). T cells were directly stimulated with plate-bound anti-CD3 and anti-CD28 antibodies (mouse anti-human, 1 μg/ml each, BD) in a time kinetic fashion (20 h, 40 h). Next, the supernatants were cleared by centrifugation and measurements were performed according the manufacturer's instructions using 100 μl cell culture supernatants.

### Statistical analysis

The results were expressed as mean ± standard deviation of at least three independent experiments. The data were analyzed using the SigmaPlot software (Systat Software, Erkrath, Germany). Differences between groups were examined for statistical significance using the Student's t test. A value of *p* < 0.05 was considered as statistically significant.

## SUPPLEMENTARY TABLE



## Abbreviations

15-LOX2: 15-lipoxygenase-2
B2m: b2-microglobuline
ccRCC: clear cell RCC
CFSE: carboxyfluorescein succinimidyl ester
DC: dendritic cells
ENO1: α-enolase
FAC: Functional annotation cluster
GAPDH: glyceraldehyde 3-phosphate dehydrogenase
GO: gene ontology
HIF: hypoxia-inducible factor
HPRT: hypo-xanthine-guanine phosphoribosyl-transferase
IL: interleukin
IPS: internal protein standard
MDSC: myeloid-derived suppressor cells
MMP: matrix metalloproteinase
NK: natural killer
PBMC: peripheral blood mononuclear cells
PPIA: peptidylprolylisomerase A
RCC: renal cell carcinoma
MnSOD: manganese superoxide dismutase
TAM: tumor-associated macrophages
TKI: tyrosine kinase inhibitors
UBE2N: ubiquitin-conjugating enzyme E2 N
VEGF: vascular endothelial growth factor
VHL: von Hippel Lindau
wt: wild-type
