# Supplementary material for: VHL-dependent alterations in the secretome of renal cell carcinoma: Association with immune cell response?
Source: Oncotarget. 2015 Oct 12;6(41):43420–37. doi: 10.18632/oncotarget.5560 (PMC4791241; doi:10.18632/oncotarget.5560)
Supplement: Supplementary file 1 [file oncotarget-06-43420-s001.pdf]

SUPPLEMENTARY TABLE

Supplementary Table S1: Primer sequences, annealing temperature and product size of different genes used in quantitative real time-PCR

gene symbol	RefGene ID	primer sequence 5’ → 3’		annealing temperature	product size (bp)
B2M	NM_004048.2	aense	CATGGAGGTTTGAAGATGCCG	60°C	233
		antisense	CTCTAAGTTGCCAGCCCTCC		
HPRT1 (TV1)	NM_000194.2	sense	GCAGACTTTGCTTTCCTTGG	60°C	101
		antisense	CTGGCTTATATCCAACACTTCG		
PPIA	NM_021130.3	sense	CCAAGACTGAGTGGTTGGATG	60°C	192
		antisense	CAGAAGGAATGATCTGGTGG		
SERPINE1	NM_000602.4	sense	CCTCTGAGAACTTCAGGATGC	60°C	153
		antisense	CCTGCTGAAACACCCTCACC		
MnSOD2	NM_001024466.1<br>NM_001024465.1<br>NM_000636.2	sense	GTTGCTGGAAGCCATCAAACG	60°C	196
		antisense	CCCCAGCAGTGGAATAAGGC		
UBE2N	NM_003348.3	sense	GCAACAGAGCGTCACTTCC	60°C	296
		antisense	GCTGGGTTTCCTTGATGATCC		
